# Clinical case study

**Published:** 2013

**Authors:** Irfan Jeeva, Aditi Das, Andy Cassels-Brown

**Affiliations:** Ophthalmology Specialist Registrar; Ophthalmic Public Health Trainee; Consultant Ophthalmologist Leeds University, Leeds, UK.

A 35-year-old man presented at our eye clinic with a 2-day history of a red, sore and watery right eye. He had visited Cameroon 4 months priorto presentation.

**Figure 1.**
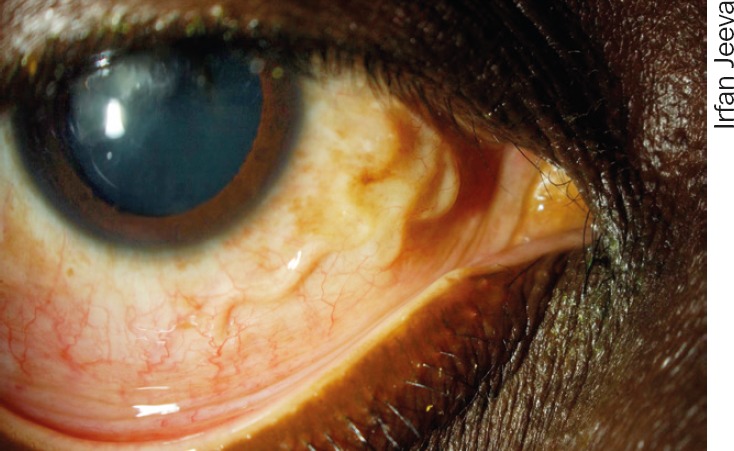


Examination of the right eye revealed an injected conjunctiva and a coiled, mobile and translucent worm in the sub-conjunctival space (Figure 1). A diagnosis of loiasis was made on the basis of clinical examination, parasitológica! analysis, a full blood count (which revealed eosinophilia) and a blood film (which showed microfilaria).

Removal of the worm *(Loa loa)* was attempted using an aseptic technique and minimal illumination. A sub-conjunctival injection of 2% lignocaine and 1:100,000 dilution of adrenaline was used to anaesthetise the eye and a 2 cm horizontal conjunctival incision was made. Despite multiple attempts to grasp the worm with forceps, it could not be extracted due to its slippery exterior. Gentle cautery was applied to seal the space around the worm and facilitate removal. Topical antibiotic was then applied and the conjunctiva closed with 6/0 vicryl. Within a week, the patient's ocular symptoms improved.

*Loa loa* is a filarial nematode with a predilection for ocular tissues. With increasing international travel it is important that ophthalmologists become familiar with the various ocular presentations of infectious diseases, which untreated can cause serious morbidity and mortality.

